# Vanillin Has Potent Antibacterial, Antioxidant, and Anti-Inflammatory Activities In Vitro and in Mouse Colitis Induced by Multidrug-Resistant *Escherichia coli*

**DOI:** 10.3390/antiox13121544

**Published:** 2024-12-17

**Authors:** Jiaxue Wang, Wei An, Zhenlong Wang, Ya Zhao, Bing Han, Hui Tao, Jinquan Wang, Xiumin Wang

**Affiliations:** 1 Institute of Feed Research, Chinese Academy of Agricultural Sciences, Beijing 100081, China; wangjiaxue@nwafu.edu.cn (J.W.); 82101222034@caas.cn (W.A.); 82101225430@caas.cn (Y.Z.); hanbing02@caas.cn (B.H.); taohui@caas.cn (H.T.); wangjinquan@caas.cn (J.W.); 2 Key Laboratory of Feed Biotechnology, Ministry of Agriculture and Rural Affairs, Beijing 100081, China; 3 College of Grassland Agriculture, Northwest A&F University, Yangling 712100, China

**Keywords:** vanillin, *Escherichia coli*, antibacterial, antioxidant, anti-inflammatory

## Abstract

A large number of cases of infectious colitis caused by multidrug-resistant (MDR) bacteria, such as *Escherichia coli*, can result in colon damage and severe inflammation. Vanilla, a widely utilized flavor and fragrance compound, is extensively used in various food. However, the effect of vanilla on MDR *E. coli*-induced infectious colitis has received less attention. In this study, the antibacterial activity of vanillin against MDR *E. coli* and other bacteria was determined by the microtiter broth dilution method. The antioxidant and anti-inflammatory capacity of vanillin was assessed in lipopolysaccharide (LPS)-stimulated RAW 264.7 cells and MDR *E. coli*-induced mouse colitis. The results demonstrated that vanillin exhibited potent antibacterial activity against various strains of MDR *E. coli*, *Salmonella*, and *Staphylococcus aureus*, with a minimal inhibitory concentration (MIC) of 1.25–2.5 mg/mL and a minimum bactericidal concentration (MBC) of 5–10 mg/mL; it effectively inhibited cell division in *E. coli*. Vanillin also displayed remarkable antioxidant activity by suppressing the levels of malondialdehyde (MDA), superoxide dismutase (SOD), and reactive oxygen species (ROS) in LPS-stimulated RAW 264.7 cell; it significantly reduced the production of inflammatory mediators including nitroxide (NO), tumor necrosis factor α (TNF-α), interleukin 6 (IL-6), and interleukin 1β (IL-1β), while increasing interleukin 10 (IL-10). In an MDR *E. coli*-induced mouse colitis model, vanillin effectively inhibited inflammation by suppressing inflammatory cytokines, mitogen-activated protein kinase (MAPK), and nuclear factor κ-B (NF-κB) cell signaling pathway activation; it ameliorated changes in intestinal microflora characterized by decreased Firmicutes richness alongside increased Bacteroides richness, rebalancing the dysbiosis caused by *E. coli*. These findings highlight the potential pharmacological applicability of vanillin as a promising bioactive molecule for treating infectious colitis.

## 1. Introduction

Infectious colitis is caused by bacteria, viruses, or parasites and can affect individuals of all age groups, with incidence increasing as one becomes older [1,2]. It is important to note that infectious colitis differs from chronic colitis, such as ulcerative colitis and Crohn’s disease, which may be associated with genetic or immune system abnormalities. A large number of cases of infectious colitis are caused by bacterial pathogens, particularly food-borne bacteria [3,4]. Multidrug-resistant (MDR) Gram-negative bacteria such as *Escherichia coli* and *Salmonella* are among the most common causative agents of infectious colitis due to their characteristic outer membrane lipopolysaccharide (LPS), which plays a major role in inducing colitis. Recent studies have demonstrated that infectious colitis can lead to colonic inflammation, hemorrhage, or hemolytic uremic syndrome, thus contributing significantly to morbidity and mortality worldwide [5].

The treatment for infectious colitis typically involves the administration of antibiotics, which can result in the development of antibiotic resistance—a global health challenge [3]. In recent years, considerable attention has been paid to plant extracts (such as phenols, flavonoids, alkaloids, quinones, etc.) due to their potential antibacterial and anti-inflammatory properties, as well as their low cytotoxicity and reduced likelihood of bacterial resistance development [6,7,8,9]. Vanillin, a phenolic aldehyde, is an active ingredient found in vanilla that has long been recognized for its health benefits—it holds significant importance as a spice and is commonly employed as an additive in various food products, such as biscuits, chocolate, milky tea, ice cream, candy, gum, and cakes [10,11,12,13]. Vanillin is utilized as a masking agent in diverse pharmaceutical formulations, a defoaming agent in certain industries, and a fragrance in cosmetics and perfumes. It is also commonly used as a pharmaceutical intermediate in the synthesis of various drugs, including berberine, the antihypertensive drug L-methyldopa, methoxy-pyrimidine, and the heart disease drug papaverine [14,15,16]. Furthermore, vanillin exhibits numerous beneficial effects on human health due to its potent antimutagenic, antimetastatic, and antidepressant activities. Even at high concentrations, vanillin demonstrates nontoxicity along with rapid absorption and the ability to cross the blood–brain barrier; thus, making it a promising candidate for treating neurological diseases [17]. The anticancer effects of vanillin have also been demonstrated, alongside its ability to potentiate antibiotics and inhibit quorum sensing (QS) [18,19,20]. Additionally, it was shown that vanillin exhibits potent scavenging activity on reactive oxygen species (ROS) and protective properties against oxidative damage [21,22,23].

Several studies have demonstrated the ability of vanillin to enhance the antibacterial properties of polyvinyl alcohol–chitosan hydrogel dressings [24,25,26]. Vanillin derivatives prevent the growth of Gram-positive bacteria (including *Micrococcus luteus* and *Bacillus subtilis*) and fungi (such as *Candida krusei*, *Paecilomyces variotii*, *Penicillium funiculosum*, etc.) [27]. Vanillin derivatives with high lipophilicity inhibit the growth of Gram-positive bacteria, including *S. aureus*, methicillin-resistant *S. aureus* (MRSA), and *Mycobacterium tuberculosis* H37Ra by permeating or disrupting their cell membranes [28]. Vanillin can regulate antibiotic resistance in *S. aureus* and *Staphylococcus epidermidis* by inhibiting efflux pump function, which is an important factor in biofilm maturity [29,30,31]. Additionally, it exhibits QS inhibition by reducing the relative mRNA expression of selected QS mechanism genes, potentially positively impacting virulence factor production and other properties, such as motility [32]. Moreover, it was found that vanillin inhibited the respiration of *E. coli*, suppressed LPS-stimulated inflammatory response in THP-1 cells, and effectively attenuated LPS-stimulated mastitis in pregnant BALB/c mice [33,34]. Wu et al. demonstrated that the oral administration of vanillin effectively prevented trinitrobenzene sulfonic acid-induced colitis by regulating the nuclear factor κ-B cell (NF-κB) signaling pathway. Another study revealed that vanillin attenuated KBrO_3_-induced depression in mice, attributing to its antioxidant and anti-inflammatory activity [35,36]. Furthermore, it has been observed that treatment with vanillin significantly suppressed the upregulation of tumor necrosis factor α (TNF-α), interleukin 6 (IL-6), and interleukin 1β (IL-1β) expression induced by KBrO_3_ exposure [37,38]. Additionally, it counteracted the decreased activity of multiple enzymes (Na^+^–K^+^ and Mg^2+^-ATPases acetylcholinesterase, and butylcholinesterase) caused by KBrO_3_, thereby alleviating neuroinflammation in BV-2 microglia. However, there have been no studies regarding whether vanillin could influence infectious colitis.

The present study aimed to evaluate the antibacterial activity against MDR *E. coli* and other bacteria, as well as anti-inflammatory effects in vitro. Additionally, we investigated the therapeutic potential of vanillin on mouse colitis induced by pathogenic MDR *E. coli* and its impact on intestinal microbial flora in vivo.

## 2. Materials and Methods

### 2.1. Materials

The MDR *E. coli* CVCC195 strain and other bacteria were stored in our laboratory. Vanillin (catalog number: V8080-25g) was purchased from Beijing Solarbio Science & Technology Co., Ltd. (Beijing, China), with a purity of ≥99%, as determined by high-performance liquid chromatography (HPLC); it was dissolved in anhydrous ethanol (solubility: ≥0.4 g/mL) and then subsequently diluted with Phosphate Buffered Saline (PBS) or Dulbecco’s Modified Eagle Medium (DMEM). *E. coli* 0111: B4 LPS was obtained from Sigma (Saint Louis, MO, USA). ELISA kits for mouse IL-6 (catalog number: MM-0163M1), IL-1β (catalog number: MM-0040M1), IL-10 (catalog number: MM-0176M1), and TNF-α (catalog number: MM-0132M1) were obtained from Jiangsu Meimian Industrial Co., Ltd. (Jiangsu, China). The anti-NF-kB p65 antibody (catalog number: ab32536), anti-p38 alpha/MAPK14 antibody (catalog number: ab170099), anti-MyD88 antibody (catalog number: ab219413), and goat antirabbit IgG H&L antibody containing Alexa Fluor^®^ dye (catalog number: bs-40295G-IRDye8) were obtained from Bioss (Beijing, China). The phospho-p38 MAPK antibody (catalog number: 9211S), phospho-NF-κB p65 antibody (catalog number: 3033T), β-tubulin antibody (catalog number: 2146S), and anti-β-actin (catalog number: 4970T) antibodies were obtained from Cell Signaling Technology (Danvers, MA, USA).

### 2.2. Antibacterial Activity of Vanillin

The minimal inhibitory concentration (MIC) and minimum bactericidal concentration (MBC) values of vanillin were determined using the microtiter broth dilution method [39]. In brief, the *E. coli*, *Salmonella typhimurium*, *S. enteritidis*, and *Staphylococcus aureus* strains were cultured, until they reached the logarithmic growth phase. Subsequently, they were diluted to 10^5^ CFU/mL and added into 96-well plates (90 μL/well). Vanillin dissolved in sterile PBS was serially diluted 2-fold and added to each well (10 μL/well). PBS served as the negative control. The plates were then incubated at 37 °C for 16–18 h until visible turbidity was observed in the negative control. The MIC value of vanillin against the tested strains was determined as the lowest concentration that completely inhibited bacterial growth. The MBC value was calculated as the vanillin concentration at which a 99.9% reduction in the initial inoculum was observed. All experiments were repeated three times.

### 2.3. Effects of Vanillin on Bacterial Morphology 

The bactericidal effects of vanillin on *E. coli* were further characterized by scanning electron microscope (SEM) to visualize morphological changes [40]. *E. coli* CVCC195 was cultured until reaching the logarithmic growth stage (1–5 × 10^8^ CFU/mL) and then diluted to optical density at 600 nm (OD_600nm_) of 1 with 0.01 M PBS. Subsequently, the *E. coli* cells were treated with 4 × MIC vanillin at 37 °C for 2 h, while untreated bacterial cells served as the control. After centrifugation, the bacteria were washed three times with 0.1 M PBS and fixed with 2.5% glutaraldehyde overnight. Following washing again, the bacterial cells were fixed for 2 h in a solution containing 1% osmium tetroxide (OsO_4_). Dehydration was carried out by immersing the samples sequentially in graded ethanol solutions (50% × 1, 70% × 1, 85% × 1, 95% × 2, 100% × 2) for 15 min each time. Finally, CO_2_ drying was performed, followed by sputtering gold-palladium prior to observation under QUANTA200 SEM (FEI, Philips, Eindhoven, The Netherlands). 

### 2.4. Cytotoxicity of Vanillin 

The effect of vanillin on cell viability was assessed using the CCK-8 assay. Briefly, mouse macrophages RAW 264.7 were cultured in DMEM medium (Gibco, Grand Island, NY, USA) supplemented with 10% fetal bovine serum (Clark Bioscience, Richmond, VA, USA) at 37 °C in a humidified incubator with 5% CO_2_. The RAW 264.7 cells (2.5 × 10^4^ cells/mL) were seeded into 96-well plates and incubated for 24 h. Subsequently, the cells were treated with various concentrations of vanillin (ranging from 0.039 mg/mL to 5 mg/mL) for another 24 h. After that, each well was supplemented with 10 μL CCK8 solution (Saint-Bio, Shanghai, China). Following a 1 h incubation, the absorbance (OD) was measured at 450 nm using a microplate reader from Thermo Fisher Scientific Shier Instrument Co., Ltd. (Shanghai, China).

### 2.5. Antioxidative Effects and Anti-Inflammatory Activity of Vanillin in LPS-Stimulated RAW 264.7 Cells

#### 2.5.1. Antioxidative Effects

The RAW 264.7 cells (1 × 10^5^ cells/well, 2 mL medium/well) were cultured in a 6-well plate and exposed to various concentrations of vanillin (0.05, 0.1, 0.2, and 0.4 mg/mL) for 3, 6, 9, and 12 h. Subsequently, the cells were stimulated with 10 μg/mL LPS for 24 h. Then, the cells were collected in cold PBS, homogenized, and centrifuged at 12,000× *g* for 5 min at 4 °C. The resulting supernatant was collected to measure the antioxidant ability using the ABTS method. The superoxide dismutase (SOD) activity and intracellular malondialdehyde (MDA) levels were determined using the SOD activity assay kit (Beyotime Biotechnology, Beijing, China) and lipid oxidation assay kit (Beyotime Biotechnology, Beijng, China), respectively.

#### 2.5.2. Nitric Oxide (NO) Production

The RAW 264.7 cells (1 × 10^5^ cells/well, 2 mL medium/well) were cultured in a 6-well plate and pretreated with various concentrations of vanillin (0.05, 0.1, 0.2, and 0.4 mg/mL) for 6 h. Subsequently, the cells were stimulated with 10 μg/mL LPS for 24 h. The supernatant was collected by centrifugation (500× *g* for 5 min). The relative level of NO in mouse macrophages was measured using a NO detection kit (Beyotime Biotechnology, Beijng, China).

#### 2.5.3. Anti-Inflammatory Activity of Vanillin 

The RAW 264.7 cells (1 × 10^5^ cells/well, 2 mL medium/well) were cultured in a 6-well plate and treated with various concentrations of vanillin (0.05, 0.1, 0.2, and 0.4 mg/mL) for 6 h. Subsequently, the cells were stimulated with 10 μg/mL LPS for 24 h. After that, the culture supernatant from each well was centrifuged at 10,000× *g* for 5 min and used to detect the TNF-α, IL-6, IL-1β, and IL-10 levels with ELISA kits, according to the manufacturer’s instructions; the absorbance was then measured at 520 nm in a microplate reader. 

Similarly, the cells were cultured overnight (6 × 10^5^ cells/well, 2 mL medium/plate) and pre-treated with vanillin for 6 h, followed by challenge with LPS for an additional 24 h. The cells were lysed using Western blot analysis and IP cell lysates and centrifuged (12,000× *g*, 10 min) to obtain the supernatant. The protein concentrations in the supernatant were measured by a BCA protein assay kit (P0012, Beyotime Biotechnology, Beijng, China), then analyzed on a sodium dodecyl sulfate–polyacrylamide gel electrophoresis gel (SDS-PAGE) and were immunoblotted onto polyvinylidene fluoride (PVDF) membranes, followed by an incubation with the primary antibodies of p65, phospho-p65 (pp65), and p38 overnight at 4 °C. After washing, the blots were incubated with the peroxidase conjugated goat–rabbit or goat–mouse antibodies. Relative protein expression levels were quantified by a densitometric measurement of chemiluminescence (ECL) reaction band. β-actin and β-tubulin were used as the internal references.

### 2.6. Efficacy of Vanillin in an Infectious Mouse Colitis Model Challenged with E. coli 

#### 2.6.1. Animals and Housing

The SPF BALB/c male mice (6-week-old, 20 ± 2 g weight) were provided by Beijing Vital River Laboratory Animal Technology Co., Ltd. (Beijing, China). The animal experiment was performed in accordance with the Animal Care and Use Committee of Institute of Feed Research (IFR) at the Chinese Academy of Agricultural Sciences (CAAS), and it received approval from the Laboratory Animal Ethical Committee and its Inspection of the IFR of CAAS (IFR-CAAS20231229).

#### 2.6.2. Experimental Design

A colitis mouse model was established to evaluate the efficacy of vanillin. Briefly, MDR *E. coli* CVCC195 was cultured at 37 °C to the logarithmic phase and resuspended in PBS (1 × 10^8^ CFU/mL). The mice were randomly divided into six groups (*n* = 8), including the blank control group (control), model control group (*E. coli*), positive control group (polymyxin B (PMB)), and low-, medium-, and high-dose vanillin groups (vanillin). From day 1 to day 20, the mice in the *E. coli*, PMB, and vanillin groups received *E. coli* CVCC195 by gavage once daily (1 × 10^8^ CFU/mL, 200 μL/each). The mice in the control group were administered an equivalent volume of normal PBS at the same time each day for 20 consecutive days. After modeling, from day 21 to day 30, the mice in the PMB group were given PMB (11.8 mg/kg) by oral gavage once daily, while those in the vanillin groups received daily oral gavage of vanillin (15, 30, and 60 mg/kg). The body weight of mice was recorded at day 20 and day 30, respectively.

#### 2.6.3. Blood Biochemical Parameter and Cytokine Testing

After the experiment, blood was collected from the mice’s eye sockets and centrifuged at 8000× *g* for 15 min. The sera were collected to detect total bilirubin (TBIL), aspartate aminotransferase/alanine aminotransferase (AST/ALT), triglyceride (TG), and γ-glutamyl transferase (GGT) using a biochemical analyzer (MNCHIP, Tianjin, China). The concentrations of IL-6, IL-8, IL-10, and TNF-α in the serum samples were determined using ELISA kits, according to the manufacturer’s instructions (Jiangsu, China). 

#### 2.6.4. Western Blot Analysis 

A total of 0.02 g of mouse colon tissue was lysed with tissue lysate in an ice bath and centrifuged at 12,000× *g* for 5 min to obtain the supernatant. Subsequently, 30 μg of proteins were analyzed by SDS-PAGE and transferred onto PVDF membranes (Merck-Millipore, Darmstadt, Germany) for immunoblotting. After blocking nonspecific sites with a solution containing 5% nonfat dry milk for 1 h at room temperature, the membranes were washed three times in tris-buffered saline with Tween 20 (TBST), followed by overnight incubation at 4 °C with primary antibodies against MYD88, p65, p-p65, and p38 (dilution of 1:1000, Abcam, Cambridge, UK), according to their guidelines. Following another three washes with TBST, the blots were incubated with peroxidase-conjugated secondary antibody, such as goat antirabbit IgG H&L antibody containing Alexa Fluor^®^ dye (dilution of 1:10,000, Abcam, Cambridge, UK) for 1 h at room temperature. Finally, the blots were analyzed using a BioRad Chemi-Doc gel system. The Western blot results were analyzed and quantified using the automatic measurement function of gray value in Adobe Photoshop 2021. β-actin and β-tubulin served as internal references.

#### 2.6.5. Histopathological Assessment with Hematoxylin and Eosin (H&E) Staining

The colon tissue (3–4 mm) was taken from mice and fixed in 4% paraformaldehyde. Subsequently, the tissue was dehydrated, embedded into a paraffin wax block, sectioned to 5 μm using a microtome, and mounted onto the stain assessment slides above the biopolymer label. The slides were placed onto a hot plate at 60 °C for 2 h and stained with 1% Mayer’s HE aqueous solution. Finally, the samples were observed under a Nikon Eclipse Ts2R + FL microscope (Nikon, Tokyo, Japan, Eclipse Ci-L) [41]. According to reference [42], the histopathological score was assessed on a scale of 0 to 4 points. Grade 0 indicates no inflammatory reaction, grade 1 represents superficial inflammation and minimal mucosal hyperplasia, grade 2 signifies mild ulceration extending into the submucosa, grade 3 denotes occasional moderate hyperplasia in both mucosa and submucosa with intermittent transmural involvement, while grade 4 indicates frequent transmural marked hyperplasia with moderate to severe goblet cell loss ulcerations and crypt loss. The final histopathological score was determined by summing up these four parameters: the degree of colonic tissue lesion (inflammation), the depth of lesion, the extent of crypt destruction, and the range of lesions. 

#### 2.6.6. 16S rRNA Gene Sequencing

After euthanizing the mice, the fecal samples (≥0.5 g/each) were collected from each mouse intestines (*n* = 4), and immediately stored at −80 °C. Genomic DNA was extracted from fecal samples using the EZNA Mag-Bind Soil DNA Kit (Omega, M5635-02, San Antonio, TX, USA), and the concentration of the DNA samples was measured using the Qubit dsDNA HS Kit (Thermo, Waltham, MA, USA). The V3-V4 region of the 16S rRNA was amplified and sequenced by next-generation sequencing at Sangon Biotech Co., Ltd. (Shanghai, China). The forward primer of the V3-V4 sequence was CCTACGGGNGGCWGCAG, while the reverse primer was GACTACHVGGGTATCTAATCC. The amplification procedure was as follows: pre-denaturation at 95 °C for 3 min, 27 cycles (denaturation at 95 °C for 30 s, annealing at 55 °C for 30 s, extension at 72 °C for 30 s), followed by stable extension at 72 °C for 10 min, and finally, storage at 4 °C [43]. 

### 2.7. Statistical Analysis

All data were analyzed using GraphPad Prism 9 statistical software, and the results were presented as the mean ± SD. Statistical analysis was performed using analysis of variance, and differences between groups were assessed using the least significant difference test. *p*-values < 0.05 were considered statistically significant.

## 3. Results

### 3.1. Antibacterial Activity of Vanillin

#### The MIC and MBC Values of Vanillin

The antibacterial activity of vanillin against pathogenic bacteria was evaluated using several strains of *E. coli*, *Salmonella*, and *S. aureus*. As shown in Table 1, the MICs of vanillin against four *Escherichia* strains were 1.25 mg/mL, with the MBCs ranging from 5 mg/mL to 10 mg/mL. The MICs and MBCs of vanillin against three *Salmonella* strains were 1.25–2.5 and 5–10 mg/mL, respectively. The MIC and MBC of vanillin against MDR *S. aureus* ATCC43300 were observed to be 2.5 and 5–10 mg/mL, respectively. The result indicates that vanillin has potent antibacterial activity against some Gram-negative and Gram-positive bacteria, including MDR strains.

### 3.2. Mechanism of Vanillin Against E. coli CVCC195

**Figure 1:**
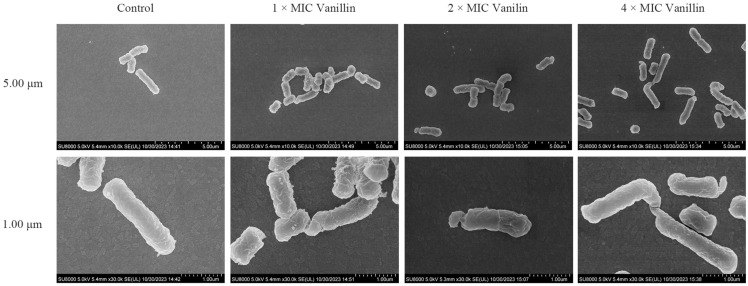
The effect of vanillin on the morphology of *E. coli* CVCC195 cells.

The *E. coli* CVCC195 cells were treated with 1×, 2×, or 4 × MIC vanillin for 2 h, and the resulting morphological changes in the cells were examined using SEM. As shown in Figure 1, the untreated *E. coli* cells exhibited an intact smooth surface and normal cell morphology. However, upon exposure to 1× or 2 × MIC vanillin, significant bacterial shrinkage along with the presence of some protrusions on the cell surfaces were observed. Treatment with 4 × MIC vanillin resulted in elongated rod-shaped bacteria, along with cell surface shrinkage and protrusion formation (Figure 1). These findings indicate that vanillin possesses inhibitory effects on the growth and division processes of *E. coli*.

### 3.3. Vanillin Exhibits Low Cytotoxicity and Potent Anti-Inflammatory Activity In Vitro

#### 3.3.1. Cell Viability of Vanillin

The cell viability of vanillin in murine peritoneal RAW 264.7 cells was assessed using the CCK-8 assay. As shown in Figure S1, the cell viability of vanillin within a concentration range of 0.04–0.32 mg/mL exceeded 100%, indicating no cytotoxicity towards mouse macrophages at a dose below 0.32 mg/mL. The cell viability of vanillin at a concentration of 0.64 mg/mL was 90%; at concentrations ranging from 1.25 to 5.0 mg/mL, the cell viability of vanillin ranged from 25% to 0%, indicating significant cytotoxicity at higher concentrations. Thus, the nontoxic concentrations (0.05, 0.10, 0.20, and 0.40 mg/mL) of vanillin were selected for subsequent cellular experiments. 

#### 3.3.2. Effect of Vanillin on Antioxidant Activity

**Figure 2:**
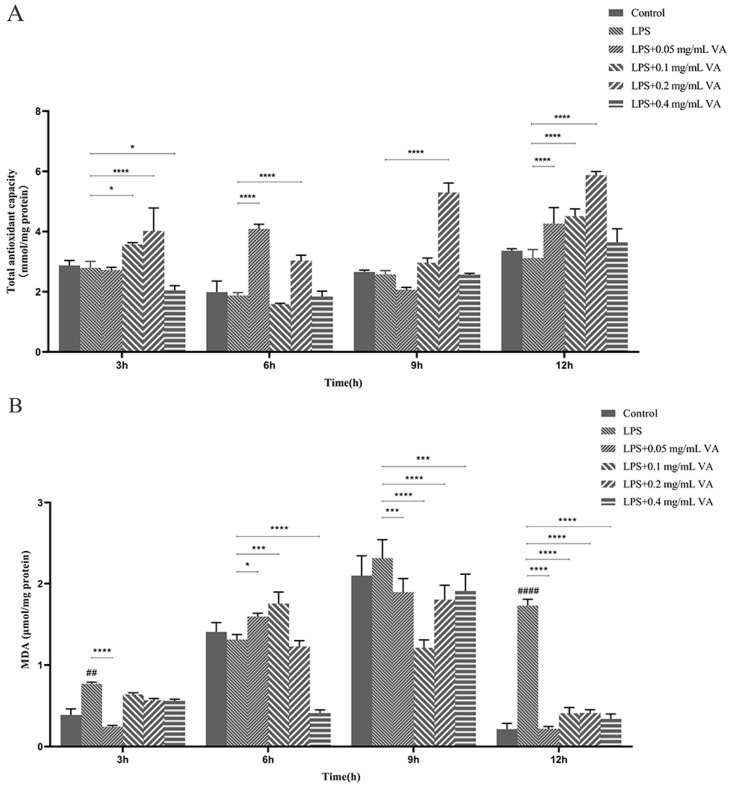
Effects of vanillin on relative production of total antioxidant capacity and MDA in RAW 264.7 cells stimulated by LPS. Mouse macrophages were divided into 6 groups: CON group, LPS group, LPS + 0.4 mg/mL vanillin, LPS + 0.2 mg/mL vanillin, LPS + 0.1 mg/mL vanillin, and LPS + 0.05 mg/mL vanillin. RAW 264.7 cells were exposed to vanillin for 3, 6, 9, and 12 h, followed by stimulation with LPS (1 μg/mL) for 24 h. The levels of related antioxidant factors were determined using commercial kits. (**A**) Effects of vanillin on relative production of total antioxidant capacity. (**B**) Effects of vanillin on relative production of MDA. The obtained data are presented as the mean ± standard deviation (SD) (*n* = 3). Significantly different from the LPS group (* *p* < 0.05, *** *p* < 0.001, and **** *p* < 0.0001). Significantly different from the control (^##^ *p* < 0.01 and ^####^ *p* < 0.0001).

To evaluate the inhibitory effect of vanillin on the production of antioxidant factors in RAW 264.7 cells stimulated by LPS, the cells were pretreated with 0.05–0.4 mg/mL vanillin for 3, 6, 9, and 12h and then stimulated with 10 μg/mL LPS for 24 h. The activity of SOD and the relative levels of total antioxidant capacity, ROS, and MDA in mouse macrophages were determined by kits. The results showed that compared with the blank control group, the total antioxidant capacity in LPS-stimulated cells decreased at 3, 6, 9, and 12 h (Figure 2A); the highest level of total antioxidant capacity was observed in the vanillin-pretreated group at a concentration of 0.20 mg/mL at each studied time point (*p* < 0.001). After pretreatment with vanillin, the total antioxidant capacity levels were increased at 12 h compared to other time points in the studied groups (Figure 2A). The content of MDA was increased in the model LPS group compared with the control group. The level of MDA in vanillin-pretreated groups was lower than that of the LPS group except for the treatment time of 6 h. The amount of MDA was significantly (*p* < 0.001) decreased to 0.24 and 0.22 μmol/mg protein compared with LPS group (1.6 μmol/mg) after pretreatment with 0.05 mg/mL vanillin for 3 h and 12 h, respectively (Figure 2B), indicating its superlative protection ability.

Additionally, LPS stimulation resulted in a decrease in cellular SOD levels. Compared with the control group, pretreatment with 0.4 mg/mL vanillin for 3 h and 6 h significantly increased SOD levels; after pretreatment with different concentrations of vanillin for 12 h, there was an elevation in SOD levels (Figure S2). However, pretreatment with different concentrations of vanillin for 9 h slightly decreased SOD activity (Figure S2). The addition of vanillin significantly decreased ROS levels (*p* < 0.001) at 3 h and 6 h, with the dosage group of 0.4 mg/mL showing the best effect (Figure S2). However, following pretreatment with vanillin and PMB for 12 h, there was an increase in the ROS levels. The results showed that vanillin effectively inhibits the production of antioxidant factors in RAW 264.7 cells stimulated by LPS.

#### 3.3.3. Effects of Vanillin on NO Production

**Figure 3:**
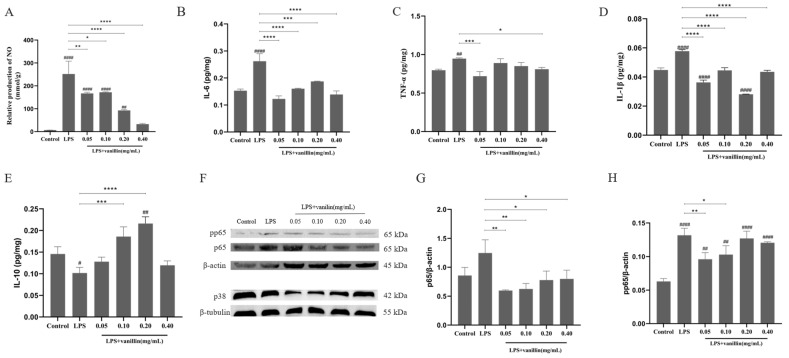
Effect of vanillin on NO production and cytokine protein expression in LPS-stimulated inflammation in RAW 264.7 cells. The experiments were divided into 6 groups: CON group, LPS group, LPS + 0.4 mg/mL vanillin, LPS + 0.2 mg/mL vanillin, LPS + 0.1 mg/mL vanillin, and LPS + 0.05 mg/mL vanillin. RAW 264.7 cells were exposed to vanillin for 6 h, followed by stimulation with LPS (1 μg/mL) for 24 h. (**A**) Effect of vanillin on relative production of NO in LPS-stimulated cells. The expressions of NO were detected by a NO detection kit. (**B**–**E**) Effect of vanillin on cytokine protein expression in LPS-stimulated RAW 264.7 cells. The expressions of IL-6 (**B**), TNF-α (**C**), IL-1β (**D**), and IL-10 (**E**) were detected by ELISA kits. The data are expressed as the mean + SD (*n* = 3). (**F**–**H**) Effect of vanillin on NF-κB/MAPK signaling pathways in mouse macrophages induced by LPS. Protein levels of p65 (**G**), p38 (**G**), and pp65 (**H**) in LPS-stimulated macrophages were detected by Western blot and analyzed by photoshop software. The values are given as the mean ± SD (*n* = 3 in each group). Significantly different from the LPS group (* *p* < 0.05, ** *p* < 0.01, *** *p* < 0.001, and **** *p* < 0.0001). Significantly different from the control (^#^ *p* < 0.05, ^##^ *p* < 0.01, and ^####^ *p* < 0.0001).

The effects of vanillin on LPS-induced inflammatory responses in RAW 264.7 cells were evaluated by quantifying the levels of NO, a proinflammatory mediator. The results showed that NO levels in LPS-treated cells were approximately 36 times that in the blank control group (*p* < 0.0001). Pretreatment with 0.05, 0.10, 0.20, and 0.40 mg/mL vanillin suppressed NO production to 2, 2, 3.5, and10 times, respectively, in LPS-stimulated cells. (*p* < 0.0001) (Figure 3A), indicating that vanillin effectively inhibits NO production in a concentration-dependent manner in LPS-stimulated RAW 264.7 cells. 

#### 3.3.4. Effects of Vanillin on Cytokines

Previous studies have demonstrated that LPS can upregulate the expression of both proinflammatory and anti-inflammatory cytokines [45,46,47]. To investigate whether vanillin modulates cytokine expression in LPS-stimulated RAW 264.7 cells, competitive ELISA was used to measure the levels of cytokines. As shown in Figure 3B–E, compared to the blank control group, the LPS-stimulated group exhibited a significant increase in IL-6 (70%), TNF-α (18%), and IL-1β (29%) levels (*p* < 0.005), while there was a significant decrease in the IL-10 level (31%) (*p* < 0.01). Following treatment with vanillin at concentrations ranging from 0.05 to 0.4 mg/mL, there was a notable reduction in TNF-α, IL-1β, and IL-6 levels (6–47%) within LPS-stimulated cells (*p* < 0.005), accompanied by a significant increase in IL-10 production (47–85%) (*p* < 0.01). These findings suggest that vanillin possesses the ability to regulate inflammatory responses by modulating the production of inflammatory cytokines in LPS-stimulated RAW 264.7 cells.

#### 3.3.5. Effects of Vanillin on LPS-Stimulated NF-κB/MAPK Pathways

To elucidate the underlying mechanism of the vanillin inhibition of LPS-activated NF-κB/MAPK pathways in RAW 264.7, the change in NF-κB/MAPK signaling molecules was assessed through Western blot analysis. As shown in Figure 3F–H and Figure S3, LPS significantly upregulated the expression and phosphorylation of NF-κB p65 protein, while downregulating p38 protein expression compared to the control group. However, pretreatment with vanillin at concentrations ranging from 0.05 to 0.40 mg/mL markedly reduced LPS-induced NF-кB p65 expression and phosphorylation; moreover, higher concentrations of vanillin (>0.05 mg/mL) led to a decrease in MAPK p38 protein expression (*p* < 0.001). It suggests that vanillin exerts inhibitory effects on LPS-stimulated NF-κB/MAPK signaling pathways. 

### 3.4. Vanillin Has Potent Efficacy in Mouse Colitis Induced by E. coli

#### 3.4.1. Anti-Inflammatory Activity of Vanillin

In the experiment, a MDR *E. coli*-induced BALB/c mouse inflammation model was established to investigate the effect of vanillin on colitis. The changes in body weight after 20 days of modeling revealed that the mice in the *E. coli*-induced model group exhibited lower body weight changes compared to those in the control group from day 20 to day 30 (Figure S4). Moreover, vanillin demonstrated superior effects on body weight when compared to PMB treatment. Specifically, the administration of 60 mg/kg vanillin significantly increased the weight changes as compared with the control group (*p* < 0.05).

**Figure 4:**
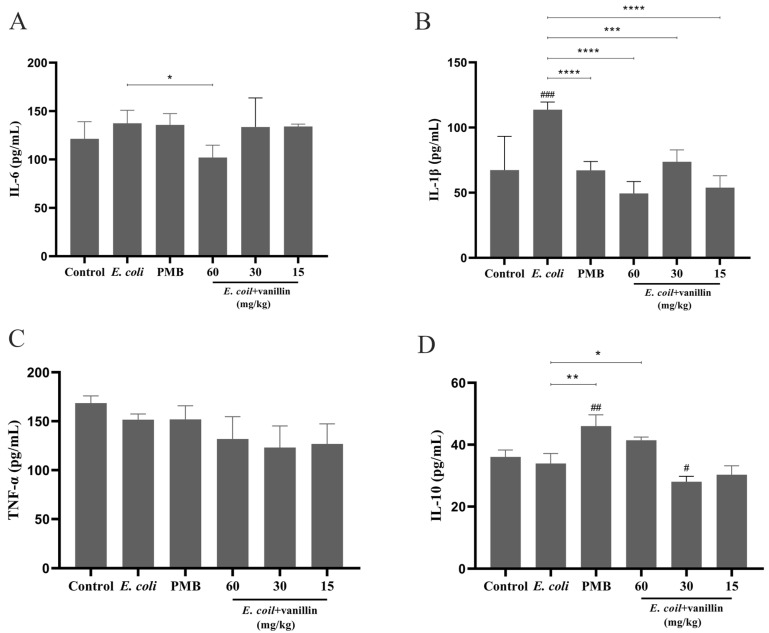
Effect of vanillin on inflammatory factors in mouse colitis induced by *E. coli*. The mice were divided into 6 groups: CON group, negative control group (*E. coli*), positive control group (11.8 mg/kg PMB), 60 mg/kg vanillin treatment group, 30 mg/kg vanillin treatment group, and 15 mg/kg vanillin treatment group. The expressions of proinflammatory factors (such as IL-6, IL-1β, and TNF-α) and anti-inflammatory IL-10 were detected by ELISA kits. (**A**) Effect of vanillin on the expression of IL-6. (**B**) Effect of vanillin on the expression of IL-1β. (**C**) Effect of vanillin on the expression of TNF-α. (**D**) Effect of vanillin on the expression of IL-10. The data are expressed by the mean ± SD (*n* = 6). Significantly different from the negative group (* *p* < 0.05, ** *p* < 0.01, *** *p* < 0.001, and **** *p* < 0.0001). Significantly different from the control (^#^ *p* < 0.05, ^##^ *p* < 0.01, and ^###^ *p* < 0.001).

The effects of vanillin on *E. coli*-induced mouse colitis were assessed by measuring inflammatory cytokine levels in serum using ELISA. As shown in Figure 4, in the model group, MDR *E. coli* significantly increased the levels of IL-6 (13%) and IL-1β (68%) in mouse serum, while reducing the level of IL-10 (6%) compared to the blank control group (*p* < 0.001), indicating a serious inflammatory response caused by MDR *E. coli*. Treatment with vanillin at doses ranging from 15 to 60 mg/kg effectively reduced the levels of IL-6 (2–26%), IL-1β (35–56%), and TNF-α (13–19%) compared to the model group, surpassing the effect of PMB at a dose of 11.8 mg/kg. Additionally, administration of 60 mg/kg vanillin concurrently increased serum IL-10 production by 22% (*p* < 0.001). However, lower doses of vanillin resulted in a decrease in IL-10 levels. No statistically significant differences were observed among the different doses of vanillin tested. These findings suggest that vanillin exerts an anti-inflammatory effect on mouse colitis by suppressing proinflammatory cytokine release and enhancing anti-inflammatory cytokine production. 

#### 3.4.2. Effects of Vanillin on Serum Biochemical Parameters

The effects of vanillin on serum biochemical parameters in *E. coli*-induced mouse colitis were assessed by a biochemical analyzer. The results demonstrated that *E. coli* significantly elevated the levels of serum TBIL, alkaline phosphatase (ALP), and AST/ALT, while reducing the levels of total cholesterol (TC), AST, and GGT compared to the control group (*p* < 0.01) (Figure S5). The administration of 11.8 mg/kg PMB resulted in a reduction in TBIL and AST/ALT levels, but it increased the ALP level. Treatment with vanillin (15–60 mg/kg) significantly reduced the serum levels of TBIL (20–38%), ALP (39–63%), and AST/ALT (2–25%) compared to the model group, indicating that vanillin exhibited a protective effect against *E. coli*-induced organ injury; meanwhile, vanillin enhanced the levels of AST, TC, and GGT (Figure S5). 

#### 3.4.3. Effects of Vanillin on MAPK and NF-κB Activation 

**Figure 5:**
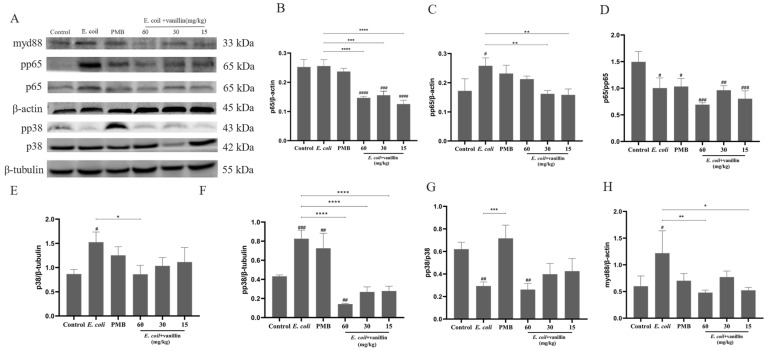
Effect of vanillin on inflammatory factors in mouse colitis induced by *E. coli*. (**A**) Effect of vanillin on MAPK and NF-κB signaling pathways. Protein level was analyzed by Western blot. (**B**) Effect of vanillin on the relative level of p65 to β-actin. (**C**) Effect of vanillin on the relative level of pp65 to β-actin. (**D**) Effect of vanillin on the relative level of p65 to pp65. (**E**) Effect of vanillin on the relative level of p38 to β-tubulin. (**F**) Effect of vanillin on the ratio pp38 to β-tubulin. (**G**) Effect of vanillin on the relative level of pp38 to p38. (**H**) Effect of vanillin on the relative level of MYD88 to β-tubulin. The values given are the mean ± SD (*n* = 3 in each group). Significantly different from the negative group (* *p* < 0.05, ** *p* < 0.01, *** *p* < 0.001, and **** *p* < 0.0001). Significantly different from the control (^#^ *p* < 0.05, ^##^ *p* < 0.01, ^###^ *p* < 0.001, and ^####^ *p* < 0.0001).

The levels of related proteins in the MAPK and NF-κB signaling pathways were assessed using Western blot analysis to investigate the impact of vanillin on inflammatory pathways. The results showed that the protein levels of p65, pp65, p38, phospho-p38 (pp38) MAPK, and MYD88 were upregulated in *E. coli*-induced mouse colitis compared to the blank control (Figure 5), indicating that *E. coli* activates both MAPK and NF-κB signaling pathways. Treatment with PMB (11.8 mg/kg) and vanillin (15, 30, and 60 mg/kg) significantly suppressed the phosphorylation of p65 and p38 as well as MYD88 production when compared to the model group (*p* < 0.001) (Figure 5). These findings suggest that vanillin exerts anti-inflammatory effects by inhibiting both MAPK and NF-κB signaling pathways. 

#### 3.4.4. Effects of Vanillin on Mouse Colon Tissues

**Figure 6:**
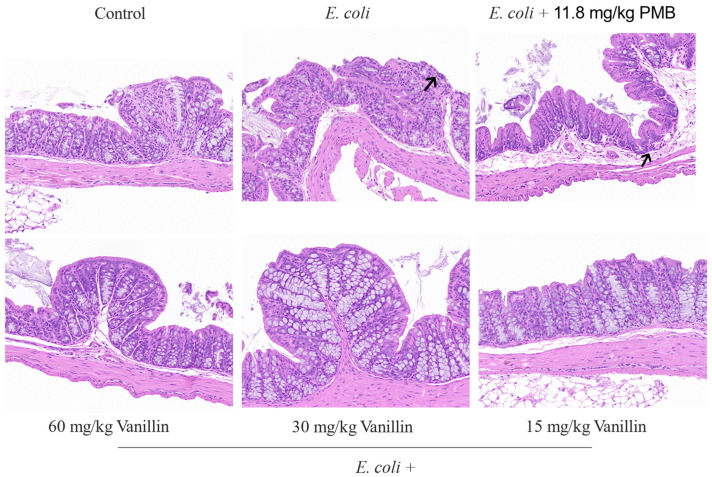
Effect of vanillin on histopathological damage of colitis induced by *E. coli*. BALB/c mice were gavaged with *E. coli* and treated with PMB (11.8 mg/kg) and vanillin (15, 30, and 60 mg/kg). Histological and pathological effects were observed under a microscope. The black arrows on the left indicates slightly increased fibrous tissue in the mucosa, while the black arrow on the right indicates slight proliferations of connective tissue in the intestinal submucosa along with an increase in submucosa space.

To evaluate the impact of vanillin on *E. coli*-induced mouse colon injury, the histological changes were examined using H&E staining. As shown in Figure 6, the colon exhibited distinct characteristic histological changes and acute injury, including necrotic epithelial cells, exfoliated intestinal mucosa, exposed intestinal glands to some extent, and slightly increased fibrous tissue in the mucosa. However, both PMB and vanillin significantly attenuated the pathological changes induced by *E. coli*. Following treatment with 11.8 mg/kg PMB, the intestinal tissue displayed intact and well-organized intestinal mucosal epithelial cells; there were slight proliferations of connective tissue in the intestinal submucosa along with an increase in submucosa space (Figure 6). Upon the administration of vanillin (15, 30, and 60 mg/kg), the integrity and arrangement of intestinal mucosa epithelial cells remained intact. The layers of intestinal mucosa, muscularis mucosa, submucosa, and muscularis were clearly delineated, while abundant goblet cells were observed within the intestinal glands of the mucosa. The histological structure appeared to be of a normal morphology following treatment with vanillin (Figure 6), while also demonstrating significant differences (Figure S6). These findings suggest that vanillin can effectively ameliorate *E. coli*-induced damage to mouse colon tissues.

#### 3.4.5. Effects of Vanillin on Intestinal Microbiota

**Figure 7:**
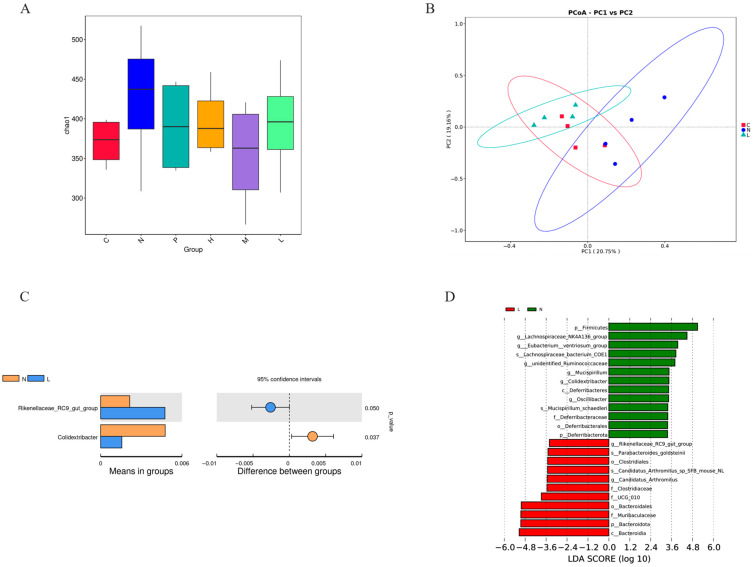
Effects of vanillin on intestinal microbiota in mice challenged with *E. coli*. (**A**) Chao1’s indices of the microbial community. C: the blank control (red); N: *E. coli* (blue); P: *E. coli* + PMB (teal); H: *E. coli +* vanillin (60 mg/kg) (yellow); M: *E. coli* + vanillin (30 mg/kg) (purple); L: *E. coli* + vanillin (15 mg/kg) (chartreuse). (**B**) Principal coordinate analysis (PCoA) of the phylum level. C: the blank control; N: *E. coli*; L: *E. coli* + vanillin (15 mg/kg). (**C**) The abundance of genera or species showing significant differences between the *E. coli* model group (N) and the low dose (15 mg/kg) of vanillin group (L). Each column represents the average abundance in each species group, showing significant differences between groups. The right figure is the confidence interval of the variation between groups. The leftmost part of each circle represents the lower limit of the 95% confidence interval, while the rightmost part is the upper limit. The center of the circle represents the difference in the average. The rightmost value is the *p* value of the significance test of variation between groups. (**D**) LDA score of the low dose (15 mg/kg) of vanillin group (L) and *E. coli* model group (N).

The fecal samples of mice were collected and subjected to 16S rRNA gene sequencing for analysis. The α diversity of the microbial community was assessed using the Chao1 index. The results demonstrated that MDR *E. coli* significantly influenced both the relative abundance and diversity of gut microbiota in mice, as evidenced by a comparison between the model group and the blank group. However, there was no statistically significant difference in microbial community diversity among groups (*p* > 0.05) (Figure 7A). 

To obtain a more intuitive representation, β diversity was combined with dimensionality reduction methods, such as principal coordinate analysis (PC). Our results demonstrated that both PMB and vanillin treatment significantly influenced the β diversity of the gut microbiota. Moreover, vanillin exhibited an ameliorative effect on *E. coli*-induced colitis by recovering microbiota similarity closer to those observed in the blank control (Figure 7B). Notably, a significant difference was observed between the model group and the low dose administration of vanillin (15 mg/kg) group (*p* < 0.05). The LDA effect size (LEfSe) showed that at the genus level, the highest LDA values in the *E. coli* model group (N) and the low dose of vanillin group (L) were Rikenellaceae_RC9_gut_group (*p* = 0.05) and Colldextribacter (*p* = 0.037) (Figure 7C,D).

**Figure 8:**
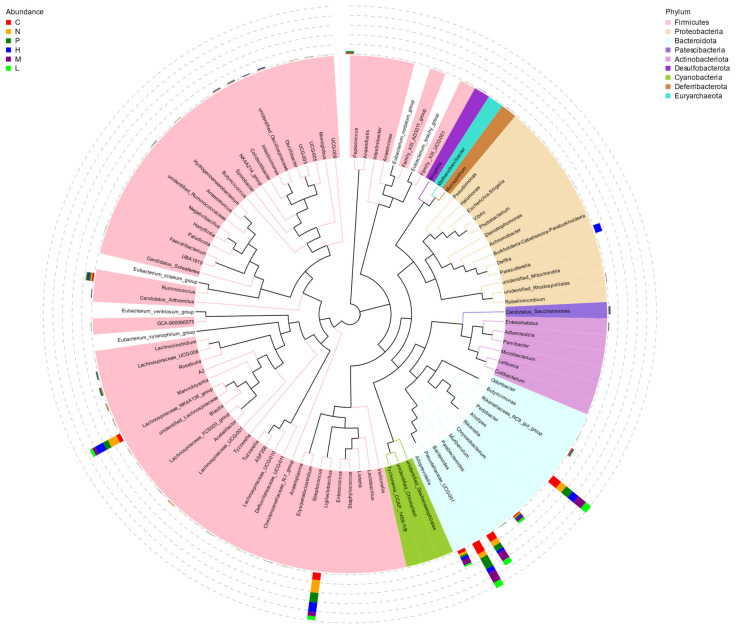
Phylogenetic tree of phylum level species. C: the blank control; N: *E. coli*; P: *E. coli* + PMB (11.8 mg/kg); H: *E. coli +* vanillin (60 mg/kg); M: *E. coli* + vanillin (30 mg/kg); L: *E. coli* + vanillin (15 mg/kg). Each branch in the phylogenetic tree represents a genus, and the length of the branch is the evolutionary distance between genera. The histogram outside the circle shows the relative proportion of reads belonging to different genera in each group. Different colors of circles represent different phyla.

**Figure 9:**
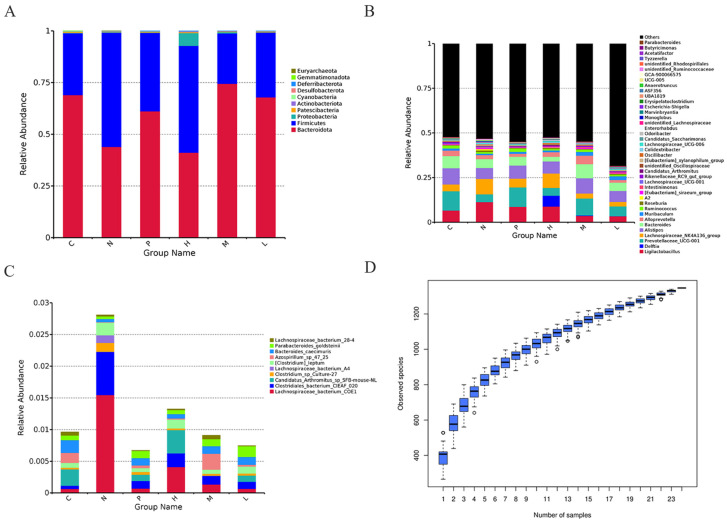
Effects of vanillin on abundance changes in intestinal microbiota in mice challenged with *E. coli*. (**A**–**C**) Abundance changes in phylum (**A**), genus (**B**), and species (**C**) in fecal microflora. (**D**) Species accumulation box diagram. C: the blank control; N: *E. coli*; P: *E. coli* + PMB (11.8 mg/kg); H: *E. coli +* vanillin (60 mg/kg); M: *E. coli* + vanillin (30 mg/kg); L: *E. coli* + vanillin (15 mg/kg).

At the phylum level, in the mice challenged with *E. coli*, Firmicutes and Bacteroides were the dominant phylum (Figure 8 and Figure 9A). After treatment with vanillin, except for the high dose group (60 mg/kg), Firmicutes and Bacteroides exhibited the highest abundance in all other groups, accounting for over 95%. Compared to the *E. coli* model group, there was a significant decrease in the abundance of Firmicutes and an increase in Bacteroides in the vanillin treatment group (*p* < 0.05). At the same time, after treatment with PMB, the microbiome of mice challenged with *E. coli* also had a drastic decrease in the proportion of Firmicutes (Figure 9).

At the genus level, the abundance of *Ligilactobacillus* and *Lachnospiracea_NK4A136*_group exhibited a decrease following the addition of vanillin; conversely, *Prevotellaceae_UCG-001*, *Alistipes*, and *Bacteroides* all demonstrated an increase after treatment with vanillin (Figure 9B) (*p* < 0.05). The abundance of *Prevotellaceae_UCG-001* and *Ruminococcus* exhibited an increase, while the abundance of *Lachnospiracea_NK4A136* and *A2* demonstrated a decrease in the PMB treatment group.

At the species level, *Lachnospiraceae_bacterium_COE1*, *Lachnospiraceae_bacterium_A4*, *Clostridiales_bacterium_CIEAF_020*, and a novel homotype acetic acid-producing bacterium (Clostridium_sp_Culture-27) were significantly decreased by vanillin. Conversely, *Bacteroides caecimuris* and *Bacteroides Goldstein* II exhibited a significant increase following the addition of vanillin (Figure 9C) (*p* < 0.05). The PMB treatment group demonstrated the lowest abundance, with a significant reduction in particularly *Lachnospiraceae_bacterium_COE1* and *Clostridium_sp_Culture-27* compared to other groups. These findings suggest that vanillin can play a key role in the clinical significance via the regulation of gut microbial dysbiosis.

## 4. Discussion

Bacterial enteropathogens are the predominant causative agents of acute infectious colitis. The emergence of MDR *E. coli* and other bacterial strains worldwide have significantly limited the efficacy of current therapeutic approaches. Therefore, there is an urgent need for novel natural alternative therapies that can effectively and safely treat infectious colitis. Vanillin, widely used as a flavoring agent and natural preservative in the food and beverage industries, has recently gained attention due to its bioactive properties, such as antibacterial, neuroprotective, anticancer, and anticarcinogenic effects. Although antioxidant and anti-inflammatory effect of vanillin has been confirmed in other inflammatory reactions, its potential role in treating infectious colitis has not been reported. This study aimed to assess the in vitro antibacterial and antioxidant activities of vanillin, as well as its anti-inflammatory potential in MDR *E. coli*-induced colitis in mice.

In this study, Gram-negative bacteria (including four *E. coli* strains and three *Salmonella* strains) and Gram-positive bacteria (such as *S. aureus* ATCC43300) were used to assess the antibacterial activities of vanillin by the microtiter broth dilution assay. Vanillin exhibited significant antibacterial activity against both Gram-negative and Gram-positive bacteria (including MDR strains), particularly demonstrating excellent antibacterial activity against the four *E. coli* strains with MIC and MBC values of 1.25 (8.21 mM) and 2.5–10 mg/mL (32.84–65.68 mM), respectively (Table 1). Previous studies have reported that vanillin possesses notable antibacterial activity against various strains of both Gram-negative and Gram-positive bacteria, including *E. coli*, *Listeria innocua*, *Aeromonas enteropelogenes*, *Pantoea agglomerans*, *Sphingobacterium spiritovorun*, *Micrococcus lylae*, *Lactobacillus plantarum*, and *L. innocua*, with the MIC values ranging from 10 to 75 mM [48]. Exposure to 1–2 × MIC vanillin resulted in the shrinkage of MDR *E. coli* CVCC195 and the formation of multiple protrusions, while treatment with 4 × MIC vanillin inhibited cell division in *E. coli* CVCC195 (Figure 1). This finding differs from a previous study in which 10–40 mM vanillin was found to inhibit cell respiration in *E. coli* MC1022 [48]. The discrepancy may be attributed to variations in the sources of *E. coli* and vanillin. 

LPS is an endotoxin present in the outer membrane of Gram-negative bacteria, serving as one of the most potent innate immune activation stimuli that triggers the production of cytokines and inflammatory mediators (such as NO and TNF-α) in macrophages [49]; therefore, LPS-stimulated RAW 264.7 macrophages can serve as a reliable model for screening anti-inflammatory drugs to investigate their impact on the signal pathway responsible for proinflammatory enzyme induction and proinflammatory cytokine production [50,51]. Similarly, in our study, *E. coli* 0111: B4 LPS was found to activate inflammatory mediators or cytokines (including IL-6, IL-1β, TNF-α, etc.) along with the protein levels of pp65, p65, and p38 in RAW 264.7 cells (Figure 3). We observed that vanillin significantly suppressed the generation of NO, IL-6, IL-1β, and TNF-α, while increasing IL-10 levels; moreover, it inhibited MAPK and NF-κB signaling pathway activation (Figure 3). These findings suggest that vanillin can regulate MAPK and the NF-κB signaling pathway to inhibit inflammatory responses, which may be beneficial for managing inflammatory diseases. It has been reported that NO functions as a cellular immune molecule, an inflammatory mediator, and a free radical. Moreover, an increase in NO concentration is positively correlated with the degree of cellular inflammation [52]. Excessive ROS can easily convert into H_2_O_2_, which easily diffuses to the cell membrane and generates highly reactive and toxic hydroxyl radicals through a heme-catalyzed Fenton reaction, eventually leading to macrophage death [53,54]. MDA is a byproduct of lipid peroxidation that serves as an indicator for evaluating lipid peroxidation [55]. Our research demonstrated that vanillin effectively suppressed the production of NO, SOD, and total antioxidant capacity in LPS-stimulated RAW 264.7 macrophages (Figure 2, Figure 3A and Figure S2). These findings indicate that vanillin exhibits remarkable anti-inflammatory and antioxidant activity in in vitro models. 

In vivo experiments revealed that vanillin effectively alleviated infectious colitis induced by MDR *E. coli* CVCC195. Histological analysis demonstrated the presence of necrotic epithelial cells, exfoliated intestinal mucosa, and increased fibrous tissue in the mucosa in MDR *E. coli*-induced colitis. As expected, treatment with vanillin ameliorated these pathological changes in colon tissues (Figure 6). The serum AST/ALT ratio is correlated with the severity of liver histopathology. Evidence suggests that an elevated AST/ALT ratio can lead to a higher grade of liver injury [56]. Our results demonstrate that in *E. coli*-induced mice, in the model group, there was an increase in the serum AST/ALT ratio; however, treatment with PMB and vanillin resulted in adjustments to the serum AST/ALT ratio, as well as TBIL and lipid profiles (such as TC) (Figure S5). This biochemical adjustment was also associated with a reduction in the levels of proinflammatory cytokines (such as IL-6, IL-1β, and TNF-α) and an increase in the production of anti-inflammatory cytokines (IL-10) (Figure 4). Furthermore, vanillin exerted its anti-inflammatory effects by inhibiting the phosphorylation of MAPK p38 and NF-κB p65 in MDR *E. coli*-induced colitis (Figure 5). Collectively, these findings indicate that vanillin confers significant protection against *E. coli*-induced damage to mouse colons. 

Intestinal microflora plays a crucial role in regulating host immunity, facilitating nutritional metabolism, and maintaining the structural integrity of the intestinal barrier [57,58]. Chronic impairment of the intestinal barrier can facilitate the translocation of LPS into circulation, leading to metabolic endotoxemia, which is commonly associated with systemic inflammation [59]. In this study, we assessed the effect of vanillin on MDR *E. coli*-induced alterations in intestinal flora in mice. Our findings revealed distinct differences in relative abundance at the phylum, genus, and species levels between the control group and vanillin-treated groups (Figure 8). At the phylum level, Cladosporium and Bacteroides accounted for approximately 95% in all groups; however, the administration of vanillin resulted in a significant decrease in Cladosporium and Firmicutes, while increasing Bacteroides abundance (Figure 8). This may be attributed to changes in the complex carbohydrates-to-protein ratio and may potentially promote enhanced intestinal tolerance [60]. At the subordinate level, MDR *E. coli* induced an imbalance in the intestinal flora; vanillin significantly increased the abundance of *Bacteroides* and *Prevotaceae_UCG-001* in mice (Figure 7A,C), thereby ameliorating colitis. On the one hand, *Prevotaceae_UCG-001* is a well-known bacterium that produces short-chain fatty acids (SCFAs) [61,62,63,64]; these active SCFAs have been demonstrated to enhance the expression of mucin-related proteins and tight junction proteins, while inhibiting the secretion of proinflammatory factors, particularly butyric acid, which serves as a vital energy substrate for colon cells and reduces the risk of pathogen infection [65,66]. On the other hand, *Lactobacillus* plays a crucial role in immune regulation and maintaining intestinal health [67]. The findings of this study are consistent with reports indicating an increase in the relative abundance of *Lactobacillus* during episodes of diarrhea [68].

## 5. Conclusions

This study demonstrated the potent antibacterial activity of vanillin against both Gram-negative and Gram-positive bacteria. Additionally, it exhibited antioxidant and anti-inflammatory effects in LPS-stimulated macrophages and MDR *E. coli*-induced mouse colitis, respectively, by suppressing ROS/MDA production, reducing inflammatory cytokines, and inhibiting the MAPK/NF-κB signaling pathway. Furthermore, vanillin was found to improve imbalanced intestinal flora in mice. These findings imply that vanillin exhibits promising potential as a novel drug candidate to the treatment of infectious colitis.

## Figures and Tables

**Table 1:** Antibacterial activity of vanillin against several pathogenic bacteria.

Strains	Vanillin	
	MIC (mg/mL, mM)	MBC (mg/mL, mM)
*Escherichia coli* CVCC1515 ^a^	1.25, 8.21	5, 32.84
*E. coli* CVCC195 ^b^	1.25, 8.21	5, 32.84
*E. coli* CVCC1560	1.25, 8.21	10, 65.68
*E. coli* 87E	1.25, 8.21	5, 32.84
*Salmonella pullorum* CVCC520	2.5, 16.42	10, 65.68
*S. typhimurium* ATCC14028 ^c^	1.25, 8.21	5, 32.84
*S. enteritidis* CVCC3377	2.5, 16.42	10, 65.68
*Staphylococcus aureus* ATCC43300 ^a^	2.5, 16.42	5, 32.84

^a^ China Veterinary Culture Collection Center (CVCC); ^b^ the MDR *E. coli* CVCC195 strain is resistant to amikacin, benzocillin, chloramphenicol, clindamycin, doxycycline, erythromycin, gentamicin, lincomycin, penicillin, streptomycin, sulfamethoxazole, and vancomycin, respectively. ^c^ American Type Culture Collection (ATCC). The *S. aureus* ATCC43300 strain is resistant to lincomycin, amoxicillin, amikacin, ampicillin, oxacillin, erythrocin, sulfisoxazole, neomycin, azithromycin, kanamycin, gentamicin, and penicillin, respectively [44].

## Data Availability

All data are contained within the manuscript and Supplementary Materials.

## Supplementary Materials

The following supporting information can be downloaded at: https://www.mdpi.com/article/10.3390/antiox13121544/s1, Figure S1: Effect of vanillin on cell viability; Figure S2: Effects of vanillin on relative production of SOD and ROS in RAW 264.7 cells induced by LPS; Figure S3: Effect of vanillin on the expression of MAPK p38 in RAW 264.7 cells induced by LPS; Figure S4: Effect of vanillin on body weight change in mouse colitis induced by MDR *E. coli*. Figure S5: Effect of vanillin on serum biochemical reaction of colitis induced by MDR *E. coli*. Figure S6: Mouse colon slice score.
