# Supplementary material for: Vanillin Has Potent Antibacterial, Antioxidant, and Anti-Inflammatory Activities In Vitro and in Mouse Colitis Induced by Multidrug-Resistant Escherichia coli
Source: Antioxidants (Basel). 2024 Dec 17;13(12):1544. doi: 10.3390/antiox13121544 (PMC11673545; doi:10.3390/antiox13121544)
Supplement: Supplementary file 1 [file antioxidants-13-01544-s001.zip › antioxidants-3326422-supplementary.pdf]

antioxidants

MDPI

Supplementary Materials

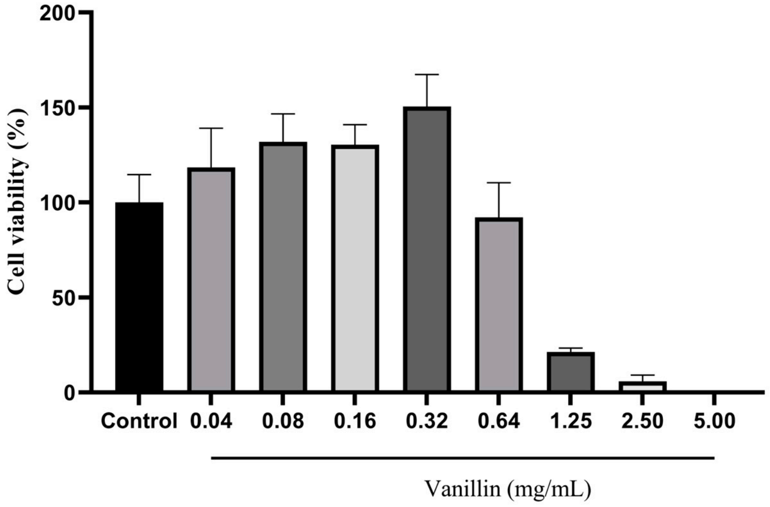

Figure S1. Effect of vanillin on cell viability. RAW 264.7 cells were cultured with different concentrations of vanillin (0.04-5 mg/mL) for 2 h, and the cell survival was determined by CCK-8 colorimetry. The data shown is the mean ± SD (n = 6).

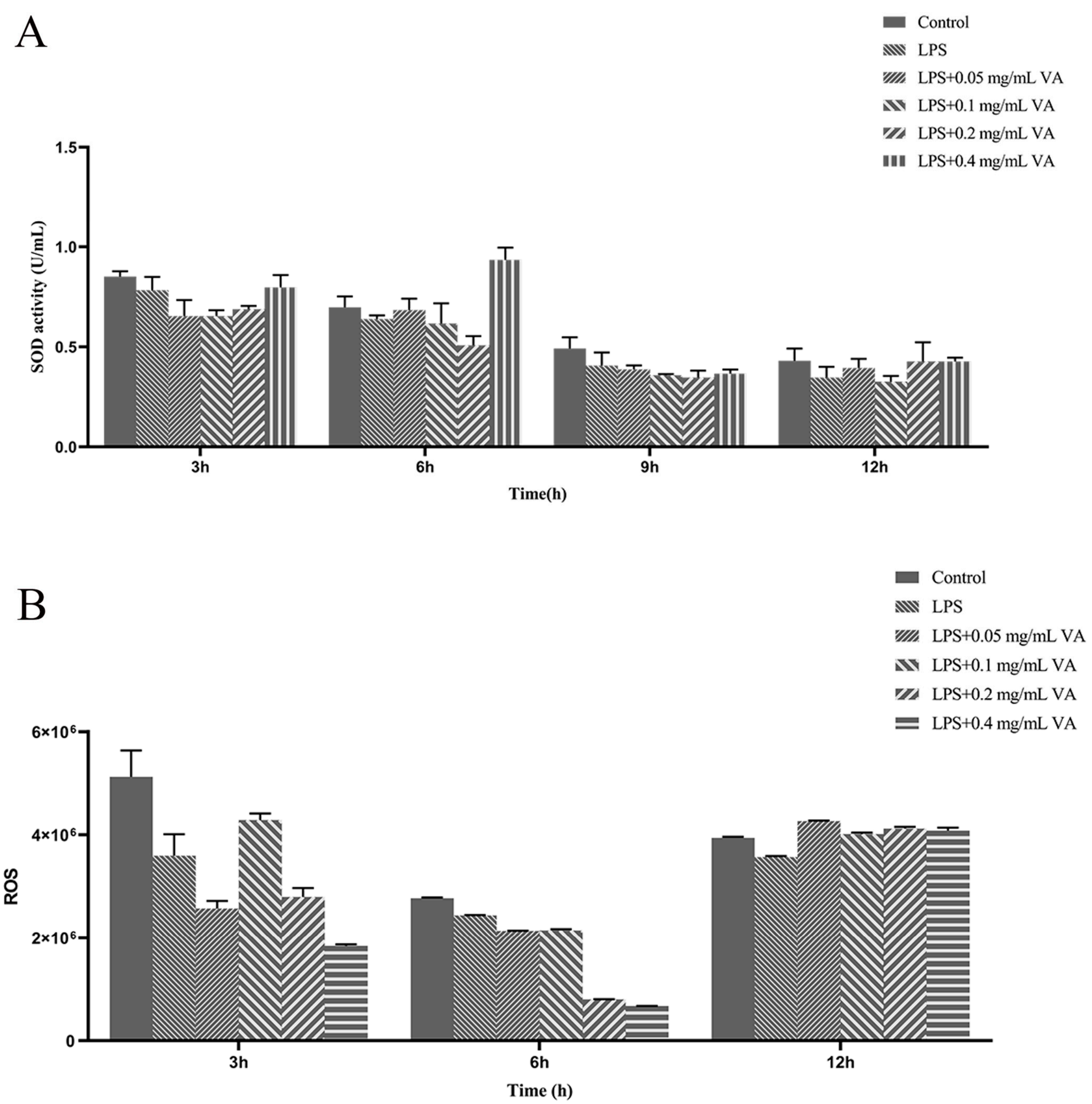

Figure S2. Effects of vanillin on relative production of SOD (A) and ROS (B) in RAW264.7 cells induced by LPS. (A) Effects of vanillin on relative production of SOD. (B) Effects of vanillin on relative production of ROS. Mouse macrophages were divided into 6 groups: CON group, LPS group, LPS+0.4 mg/mL vanillin, LPS+0.2 mg/mL vanillin, LPS+0.1 mg/mL vanillin, and LPS+0.05 mg/mL vanillin. RAW264.7 cells were cultured for 24 h; vanillin was added into the cells, followed by the addition of LPS (1 μg/mL) after 6 h. The supernatant was collected at 24 h, and the SOD activity and ROS level were determined by commercial kits. The data are expressed as mean ± SD (n=3).

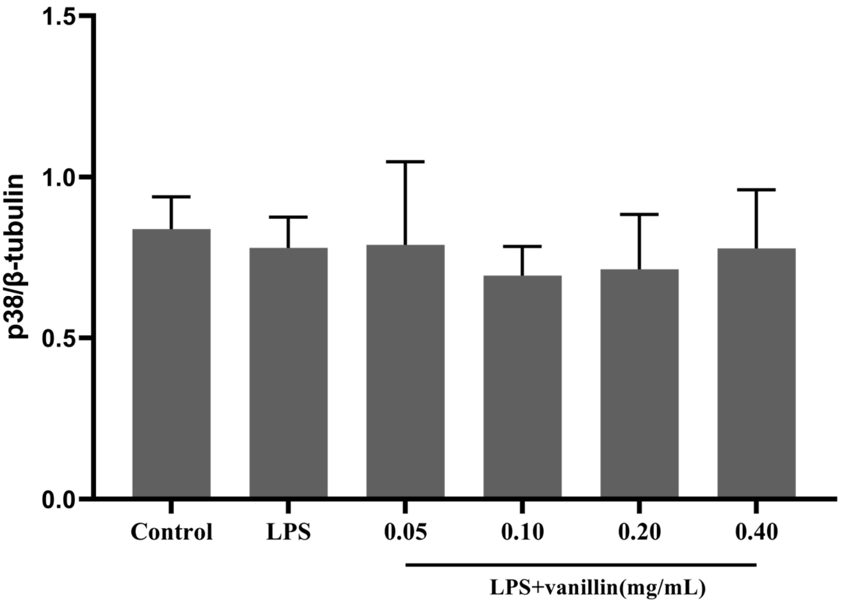

Figure S3. Effect of vanillin on the expression of MAPK p38 in RAW264.7 cells induced by LPS. Protein level was analyzed by photoshop software. The values are given as the mean ± SD (n=3 in each group).

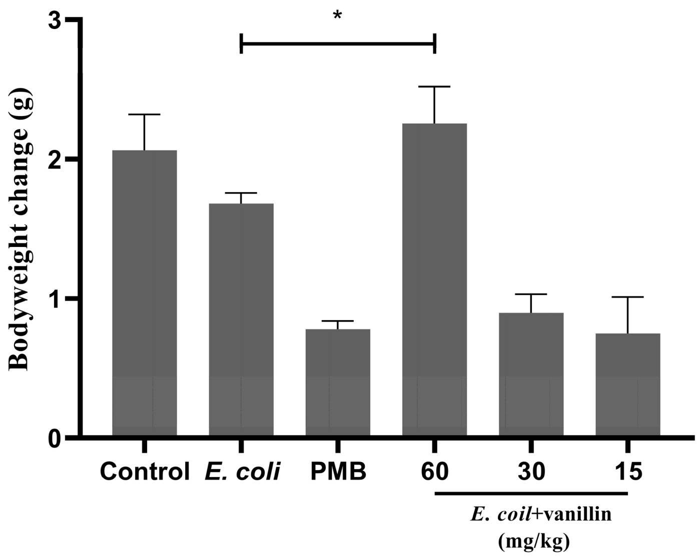

Figure S4. Effect of vanillin on body weight change in mouse colitis induced by MDR *E. coli*. BALB/c mice were gavaged with MDR *E. coli* and treated with PMB (11.8 mg/kg) and vanillin (15, 30, and 60 mg/kg). (*$p < 0.05$).

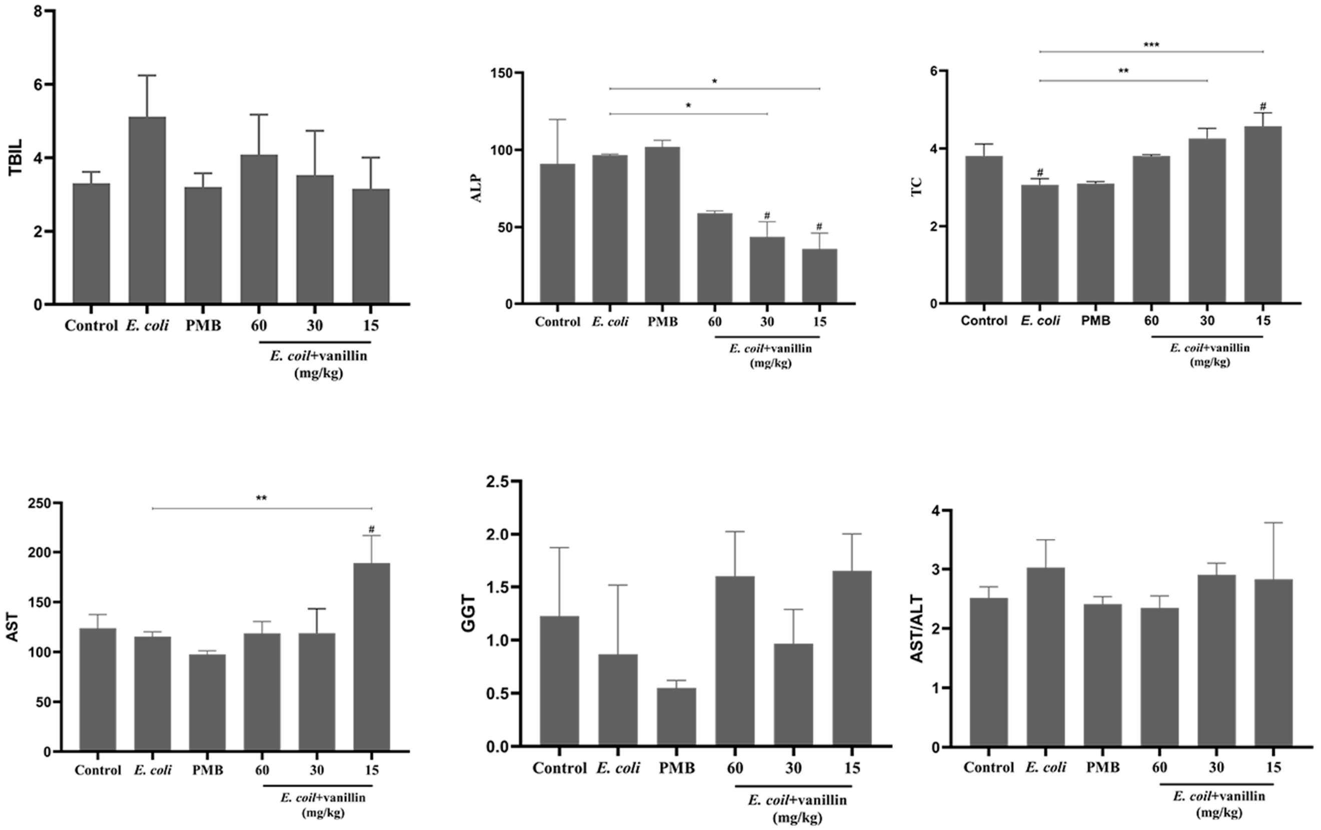

Figure S5. Effect of vanillin on serum biochemical reaction of mouse colitis induced by MDR *E. coli*. The mice were divided into 6 groups: CON group, negative control group (*E. coli*), positive control group (PMB), 60 mg/kg vanillin treatment group, 30 mg/kg vanillin treatment group, and 15 mg/kg vanillin treatment group. TBIL: total bilirubin; ALP: alkaline phosphatase; TC: total cholesterol; AST: aspartate transaminase; GGT: gamma-glutamyltransferase; AST/ALT: alutamic oxaloacetic transaminase/glutamic pyruvic transaminase. Significantly different from the negative group ($^{*}p < 0.05$, $^{***}p < 0.001$ and $^{****}p < 0.0001$). Significantly different from the control ($^{\#}p < 0.05$).

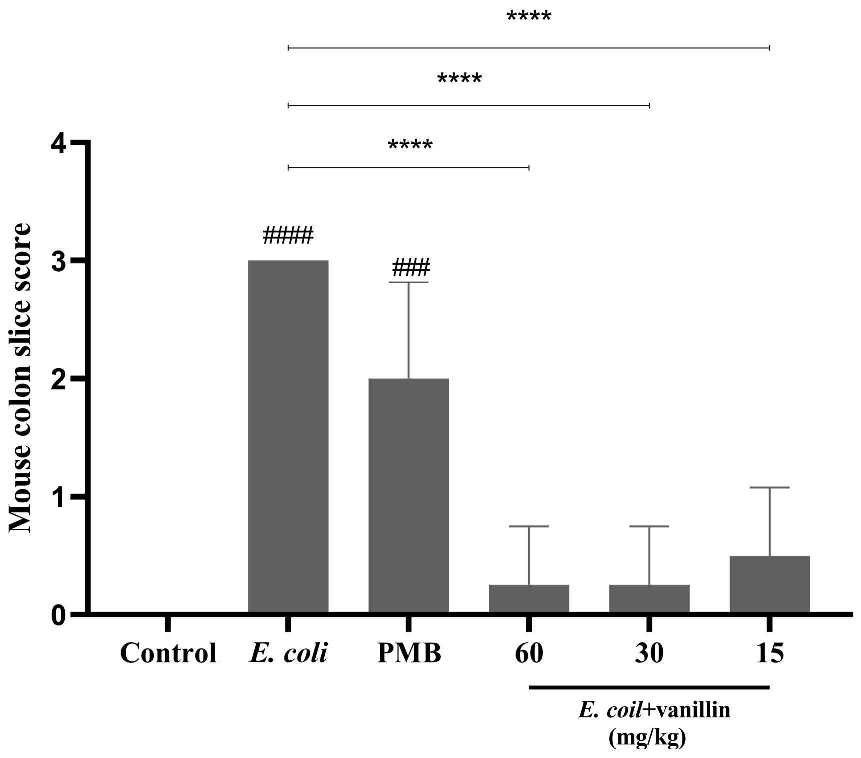

Figure S6. Mouse colon slice score. BALB/c mice were gavaged with MDR *E. coli* and treated with PMB (11.8 mg/kg) and vanillin (15, 30, and 60 mg/kg). Histological and pathological effects were observed under a microscope. According to reference [42], the histopathological score was assessed on a scale of 0 to 4 points. Significantly different from the negative group ($^{****}p < 0.0001$). Significantly different from the control ($^{###}p < 0.001$ and $^{####}p < 0.0001$).

Disclaimer/Publisher's Note: The statements, opinions and data contained in all publications are solely those of the individual author(s) and contributor(s) and not of MDPI and/or the editor(s). MDPI and/or the editor(s) disclaim responsibility for any injury to people or property resulting from any ideas, methods, instructions or products referred to in the content.
